# The MonAMI project: the social benefits of telecare

**Published:** 2012-06-15

**Authors:** Jacqueline Damant, Martin Knapp, Sarah Watters, Derek King, Maggie Ellis, Paul Freddolino

**Affiliations:** Personal Social Services Research Unit, London School of Economics, UK; Personal Social Services Research Unit, London School of Economics, UK; Personal Social Services Research Unit, London School of Economics, UK; Personal Social Services Research Unit, London School of Economics, UK; Personal Social Services Research Unit, London School of Economics, UK; School of Social Work, Michigan State University, USA

**Keywords:** telecare, older people, accessibility, disability, quality of life

## Abstract

**Introduction:**

There are widespread concerns around the accessibility of mainstream technology which remains underdeveloped for the needs of many older people 1. The Mainstreaming on Ambient Intelligence (MonAMI) project 2, funded under the EU FP6 framework in the area of e-Inclusion, developed an open technology platform and series of ‘telecare’ services designed to be accessed using mainstreamed devices (e.g. mobile telephones, personal computers). A field trial was conducted to evaluate the technology in the homes of disabled older people in three communities across Europe.

**Aims:**

The primary aim of the MonAMI trial was to evaluate the acceptability and usefulness of the services in terms of whether they enable older people with needs to live independently and maintain a good quality of life. The secondary aim was to validate the technical and economic feasibility of delivering telecare services, using an open platform, in living community contexts.

**Methods:**

The MonAMI system was evaluated in a three-month field trial in the cities of Kosice (Slovakia), Stockholm (Sweden) and Zaragoza (Spain). Trial participants (users) included people over 65 years, with a varying range of physical, cognitive and sensorial impairments. In each of the users’ homes a bouquet of alert, home monitoring and home control services were installed and configured to users’ individual needs and lifestyle. Face-to-face, semi-structured interviews were held at baseline and at post-trial follow-up, collecting data on users’ level of independence, health and wellbeing status, social networking, perceptions of safety, and acceptance of the services. Significance tests and regression analyses were performed to measure the impact of the services on users’ quality of life.

**Results:**

Sixty-eight users were recruited to the trial. The mean age was 78 years, 74% were women and 93% lived alone. Most users reported disabilities related to their legs and knees and the mean self-rated EQ5D-VAS3 health score was 63.8. In general, users with better self-rated independence 4 and health scores at baseline found the services more helpful compared to users with poorer ratings. However, specific analyses revealed the services had positive effects on perceptions of safety for users with a greater number of disabilities as compared to those with fewer disabilities (Odds ratio 2.51, p<0.05). Users with leg impairments conveyed feeling more energetic as compared to those without such impairments (Odds ratio 7.49, p<0.05); while users living alone confirmed that the services were helpful for their social networking5 as compared to users living with others (Odds ratio 2.49, p<0.1). Collectively, significantly more than 50% of users (70.6% p<0.001) rated the acceptability and usefulness of MonAMI services highly. Moreover many users stated that if possible, they would continue using the MonAMI system beyond the trial.

**Conclusions:**

Overall, the trial results are promising; demonstrating that the MonAMI telecare services are beneficial for older people with needs. The trial also confirmed the potential of deploying and providing continuous services using MonAMI technology across diverse health and social care systems in Europe, lending support to MonAMI’s proof of concept of an open core platform approach for delivering care services.

